# Supplementary material for: Integrative genomics sheds light on the immunogenetics of tuberculosis in cattle
Source: Commun Biol. 2025 Mar 24;8:479. doi: 10.1038/s42003-025-07846-x (PMC11933339; doi:10.1038/s42003-025-07846-x)
Supplement: Supplementary file 1 — Supplementary Information [file 42003_2025_7846_MOESM1_ESM.pdf]

Supplementary Information

Integrative genomics sheds light on the immunogenetics of tuberculosis in cattle

John F. O'Grady[1], Gillian P. McHugo[1], James A. Ward[1], Thomas J. Hall[1], Sarah L. Faherty O'Donnell[1,9], Carolina N. Correia[1,10], John A. Browne[1], Michael McDonald[1], Eamonn Gormley[2], Valentina Riggio[3,4], James G. D. Prendergast[3,4], Emily L. Clark[3,4], Hubert Pausch[5], Kieran G. Meade[1,6], Isobel C. Gormley[7], Stephen V. Gordon[2,6,8], David E. MacHugh[1,6,8*]

[1] UCD School of Agriculture and Food Science, University College Dublin, Belfield, Dublin, D04 V1W8, Ireland.

[2] UCD School of Veterinary Medicine, University College Dublin, Belfield, Dublin, D04 V1W8, Ireland.

[3] The Roslin Institute and Royal (Dick) School of Veterinary Studies, University of Edinburgh, Midlothian, EH25 9RG, UK.

[4] Centre for Tropical Livestock Genetics and Health (CTLGH), Roslin Institute, University of Edinburgh, Easter Bush Campus, EH25 9RG, UK.

[5] Animal Genomics, ETH Zurich, Universitaetstrasse 2, 8006, Zurich, Switzerland.

[6] UCD Conway Institute of Biomolecular and Biomedical Research, University College Dublin, Belfield, Dublin, D04 V1W8, Ireland.

[7] UCD School of Mathematics and Statistics, University College Dublin, Belfield, Dublin, D04 V1W8, Ireland.

[8] UCD One Health Centre, University College Dublin, Belfield, Dublin, D04 V1W8, Ireland.

[9] Present address: Irish Blood Transfusion Service, National Blood Centre, James's Street, Dublin, D08 NH5R, Ireland.

[10] Present address: Children's Health Ireland, 32 James's Walk, Rialto, Dublin, D08 HP97, Ireland.

[*] Corresponding author

Contents

Supplementary Note 1 3

Genomic and transcriptomic data generation 3

Supplementary Note 2 4

Variant remapping and strand flipping 4

Genome-wide SNP imputation 5

Supplementary Figures 6

References 19

Supplementary Note 1

Genomic and transcriptomic data generation

Genomic DNA was extracted and purified for each animal (bTB+ and bTB−) using 200 µl of whole blood and the QIAamp® DNA Blood Mini Kit (Qiagen) following the manufacturer's recommendations. Purified DNA was quantified using a NanoDrop™ One microvolume spectrophotometer (Thermo Fisher Scientific) and stored at −20°C. The extracted DNA was used for SNP genotyping using the Axiom™ Genome-Wide BOS 1 Bovine Array (Thermo Fisher Scientific), which assays 648,315 SNPs across the bovine genome. SNP array genotyping was performed by an external provider (IdentiGEN/MSD Animal Health).

Total RNA preparation was performed for each animal (bTB+ and bTB−) using the Tempus™ Spin RNA Isolation Kit (Thermo Fisher Scientific). The Tempus™ tubes were inverted to mix the contents, which were then poured into sterile 50 ml conical tubes and total RNA was isolated and purified according to the manufacturer's instructions. Following total RNA purification, an Agilent 2100 Bioanalyzer and the RNA 6000 Nano LabChip kit (Agilent Technologies, Inc.) were used to quality check and generate RNA integrity number (RIN) values for all samples, which were then stored at −80°C. Total RNA samples (bTB+ and bTB−) were used with the NEBNext® Ultra™ RNA Library Prep Kit for Illumina® (New England Biolabs) to construct sequence barcode-indexed RNA-seq libraries, which were then used for 150 bp paired-end sequencing on the NovaSeq™ 6000 Sequencing System (Illumina, Inc.). RNA-seq library preparation and sequencing were performed by an external provider (Novogene).

Supplementary Note 2

Variant remapping and strand flipping

Genomic coordinates of variants were updated from UMD3.1 to the ARS-UCD1.2[1] bovine genome assembly using liftOver implemented in the R package rtracklayer v.1.54.0[2] and only SNPs were retained that matched the positions in a file of the Affymetrix Axiom™ Genome-Wide BOS-1 array coordinates updated to ARS-UCD1.2 available from the United States Department of Agriculture National Animal Genome Research Program (USDA-NAGRP) data repository (www.animalgenome.org/repository/cattle/UMC_bovine_coordinates). Due to potential strand flipping issues, all remaining palindromic SNPs were removed. Raw SNP genotype files with updated coordinates were then filtered using PLINK v1.90b6.25 to remove animals with a genotype call rate < 0.95 and to restrict analysis to autosomal SNPs with a call rate > 0.95.

Strand mismatches or miscoding of alleles can significantly reduce imputation performance[3] and statistical power in the context of genomic association studies[4]. To identify the alleles and associated genotypes that needed to be flipped, original reference and alternative allele pairs for each SNP were first determined using the Axiom™ Genome-Wide BOS-1 Array master annotation file (www.thermofisher.com/order/catalog/product/sec/assets?url=TFS-Assets/LSG/Support-Files/Axiom_GW_Bos_SNP_1-na35-annot-csv.zip) due to issues associated with the encodings of the major and minor alleles in PLINK. Following this, the reference and alternative allele pairs of the filtered VCF file were compared to the USDA-NAGRP BOS-1 ARS-UCD1.2 reference allele file (www.animalgenome.org/repository/cattle/UMC_bovine_coordinates). If the NAGRP reference allele matched the alternative allele or the complement of the alternative allele in the filtered VCF file, the SNP and associated genotypes were flipped. Of the remapped variants, 100,888 SNPs (18.23%) were flipped due to ambiguity associated with reference and alternative allele strands. By following guidelines detailed in Winkler *et al.*[4], to verify that the strand flipping procedure was implemented correctly, the ARS-UCD1.2 reference allele frequencies of the target set were compared to 56 European animals derived from a whole genome sequence (WGS) Global Reference Panel[5] at matched genomic loci. A total of 236,325 SNPs overlapped between the two groups, and we observed a significant positive correlation between the allele frequencies present in both groups (Spearman correlation (ρ) = 0.98; $P < 2.2 \times 10^{-16}$) (**Supplementary Fig. S4**). A total of 150 SNPs present in the target and European WGS reference set were identified as potentially harbouring spurious allele frequency patterns through substantial deviation from the line of parity. These SNPs were removed from the target set and WGS Global Reference Panel. Hence, the number of SNPs remaining in the target set and the WGS Global Reference Panel prior to imputation was 553,369 and 10,282,037, respectively.

Genome-wide SNP imputation

For the imputation of the SNP-array data up to WGS scale, a global cattle reference panel was used, which comprised a total of 10,282,187 SNPs derived from n = 287 distinct animals spanning a diverse range of breeds and geographic locations (55 populations: 13 European, 12 African, 28 Asian, and two Middle Eastern). Details of how the original WGS data were processed is provided by Dutta *et al.* [5] and the quality control procedure implemented on the data set is described by Riggio *et al.* [6]. Briefly, Illumina WGS data for n = 427 animals representing global cattle breeds were aligned to the UCD-ARS1.2 genome. Animals and SNPs with a high proportion of missingness (> 25%), SNPs with a poor genotype quality (GQ) (< 25) and highly related individuals (relatedness value estimated from VCFtools v.0.1.15[7] with *-relatedness2*[8] > 0.0625) were removed leaving a total of n = 287 animals with 10,282,187 variants. The 150 genotyped SNPs with spurious allele frequency patterns were also removed from the WGS reference set prior to imputation.

The VCFtools package was used to split the reference and target data sets into 29 VCF files representing the 29 bovine autosomes. Beagle v.5.4[9] was then used to phase the reference and target data sets separately and Minimac3 v.2.0.1[10] was used with default settings to generate the reference haplotype m3.vcf files. Following this, Minimac4 v.1.03[10] was used with default parameters to impute the target genotype data set up to WGS scale and Bcftools v.1.7[11] was used to concatenate all 29 imputed VCF files, which resulted in a master imputed data set consisting of all n = 123 animals with genotypes for 10,282,037 SNPs.

Supplementary Figures

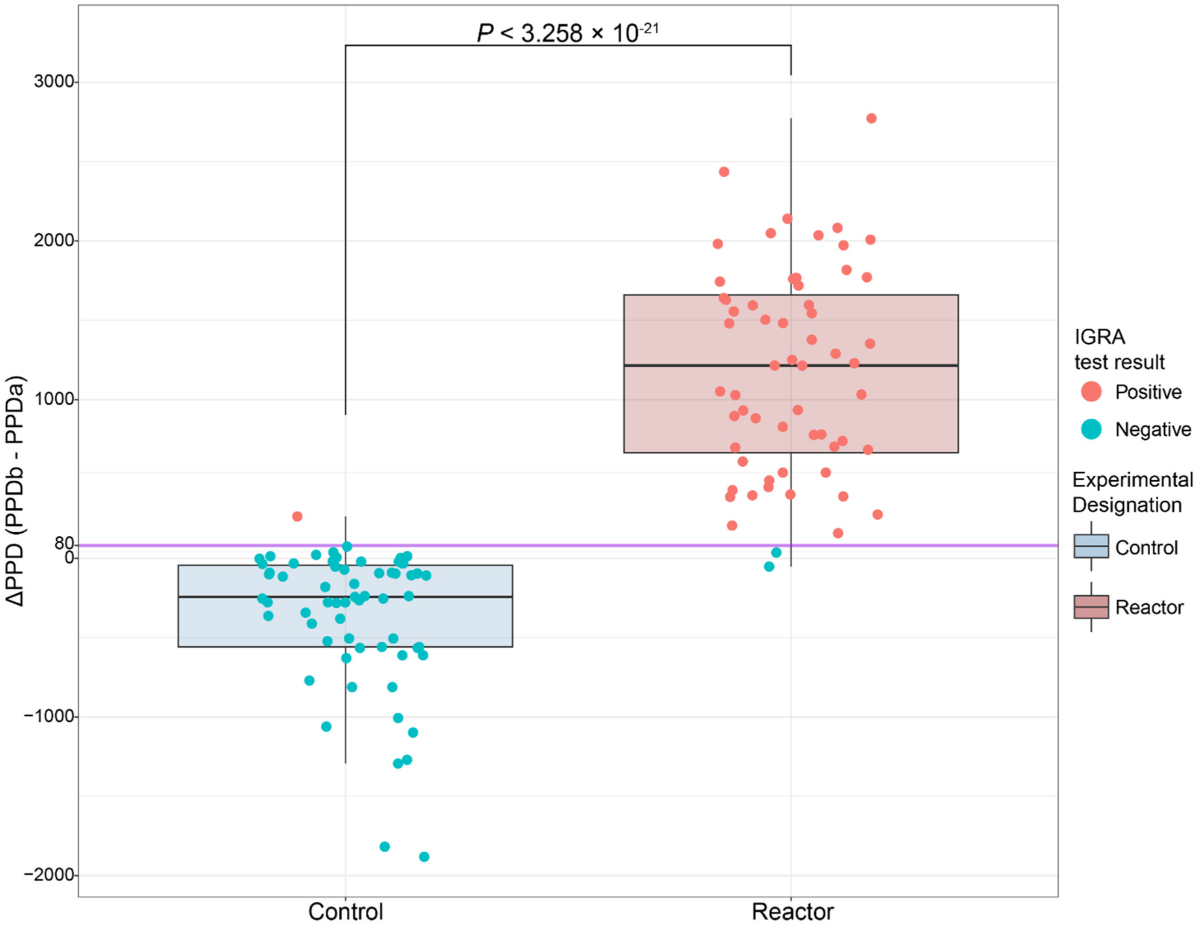

Fig. S1: IFN-γ release assay (IGRA) test results for all (*n* = 123) animals in the present study. Interferon-gamma (IFN-γ) release assay (IGRA) test results for animals designated as control (bTB−; *n* = 63) or reactor (bTB+; *n* = 60) animals. The *y*-axis denotes the Δ purified protein derivative (ΔPPD) value, calculated as PPD-bovine (PPDb) IFN-γ value minus the PPD-avian (PPDa) IFN-γ value. The purple line indicates the threshold for determining whether an animal is positive or negative for the IGRA test. Horizontal lines inside the boxes show the medians, Box bounds show the lower quartile (Q1, the 25th percentile) and the upper quartile (Q3, the 75th percentile).

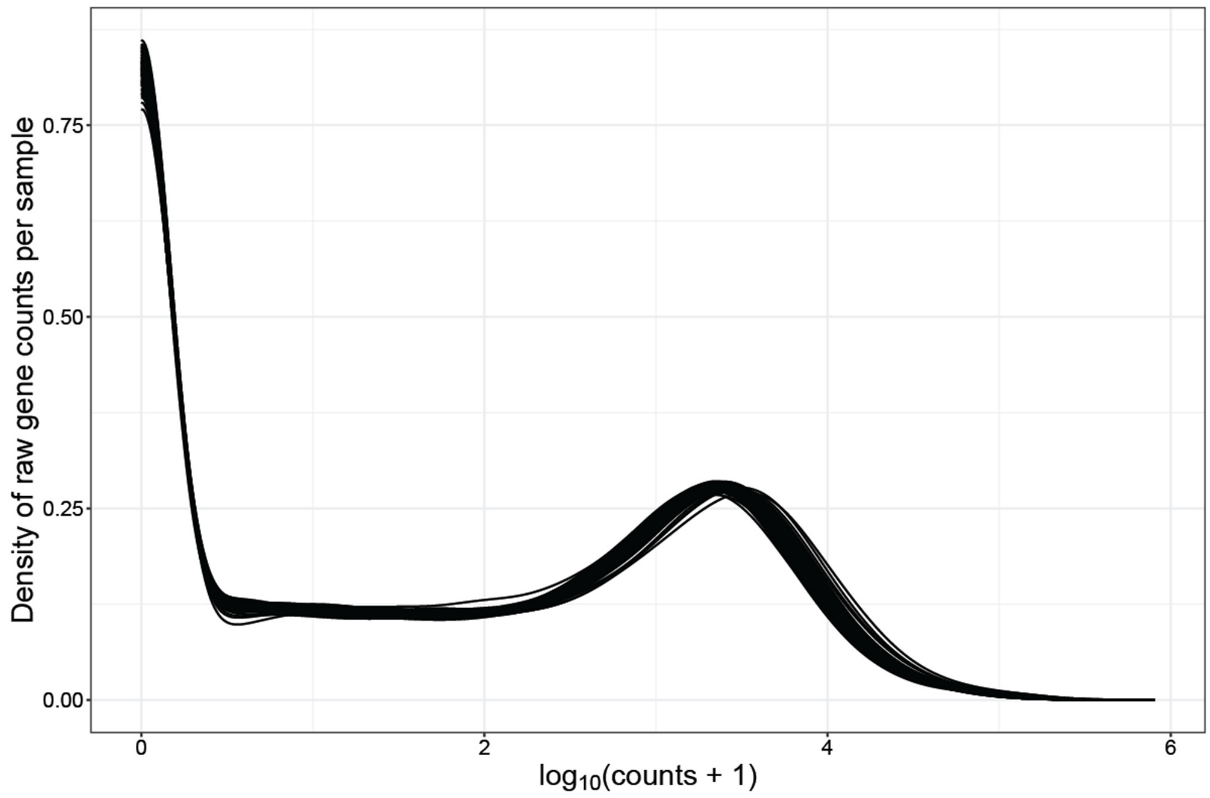

Fig. S2: Transcriptomics data quality control. Density of $\log_{10}$ gene counts per library (n = 123) prior to filtering of lowly expressed genes.

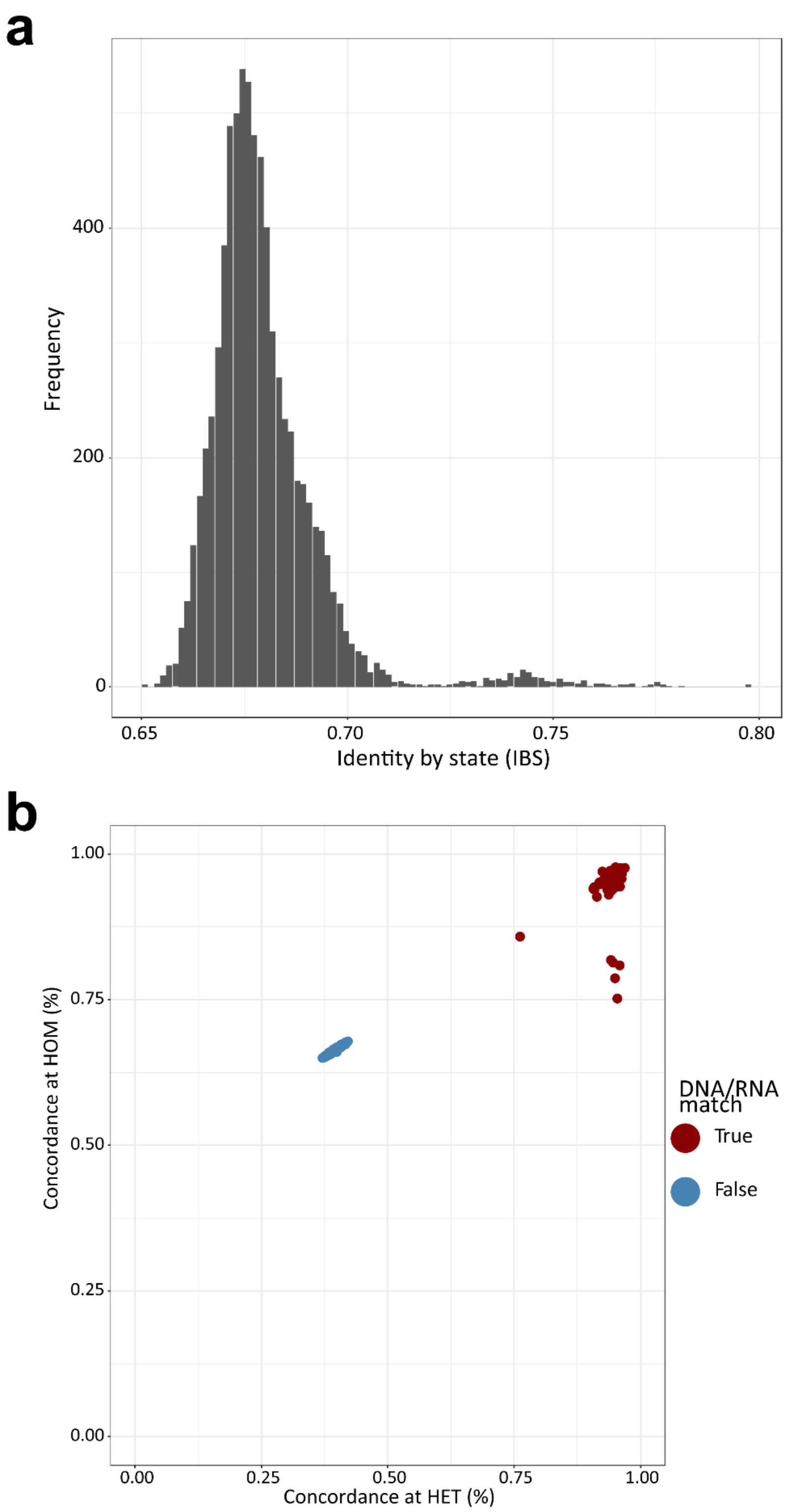

Fig. S3: Sample duplication and DNA/RNA match assessment. a Identity by state (IBS) values from PLINK[12] for all pairwise comparisons among the *n* = 123 animals in the current study using the pruned SNP data prior to imputation. **b** Match BAM to VCF (MBV)[13] outputs showing allelic consistency for heterozygous (*x*-axis) and homozygous (*y*-axis) variants, between genotype and RNA-seq sequences. Red dots indicate proper identity match of RNA-sequencing with expected genotypes. As a control, we show consistency levels for non-matching samples between biological sample T064 and all other samples (blue dots).

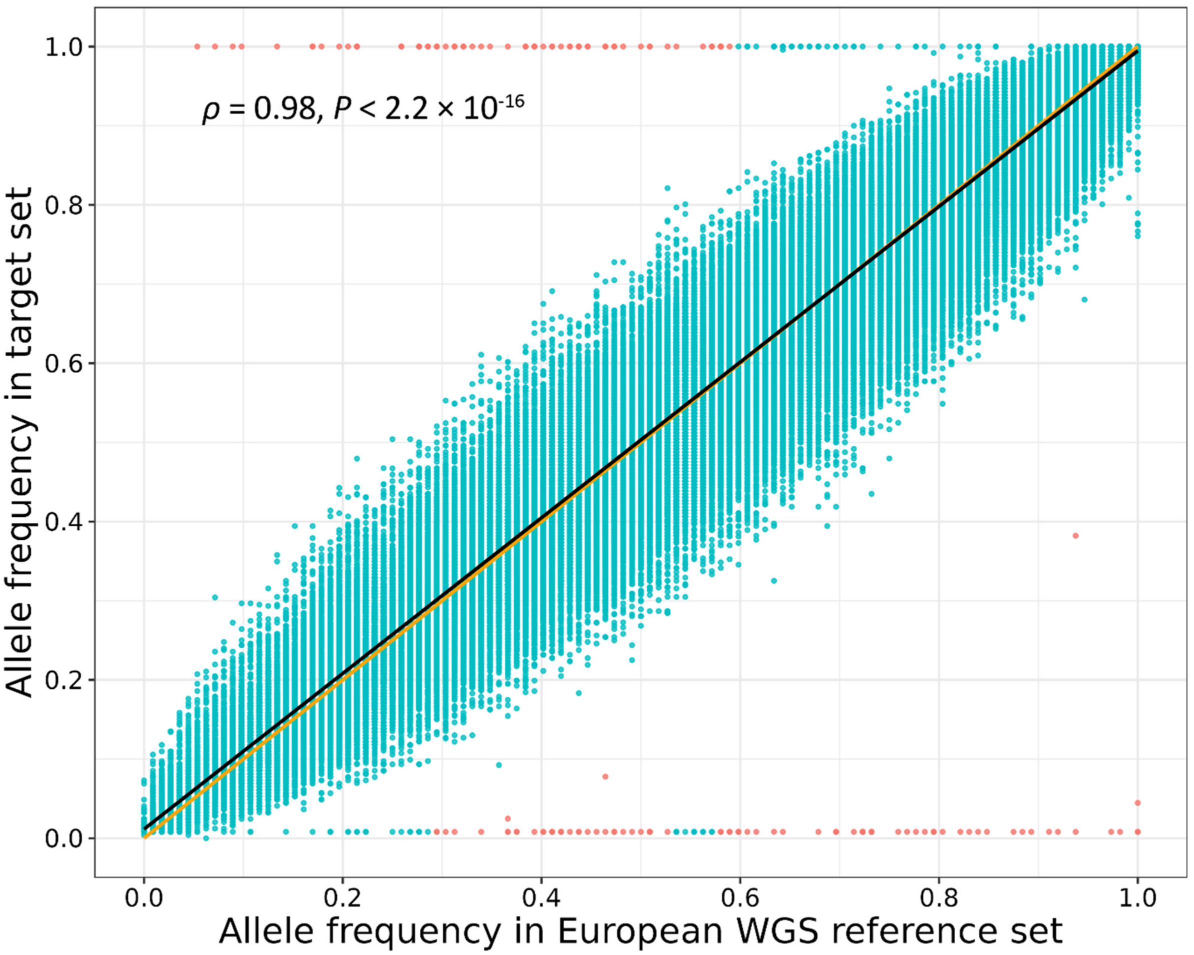

Fig. S4: Allele frequencies between the study population and European *B. taurus* cattle from the WGS reference panel. The observed (study-specific; *n* = 123) allele frequencies reported on the *y*-axis are plotted against allele frequencies derived from *n* = 56 European WGS animals on the *x*-axis at 236,325 matched genomic loci. Orange line denotes the line of parity. Black line denotes line of best fit. Red points indicate variants removed from the study population and Global Reference Panel prior to imputation.

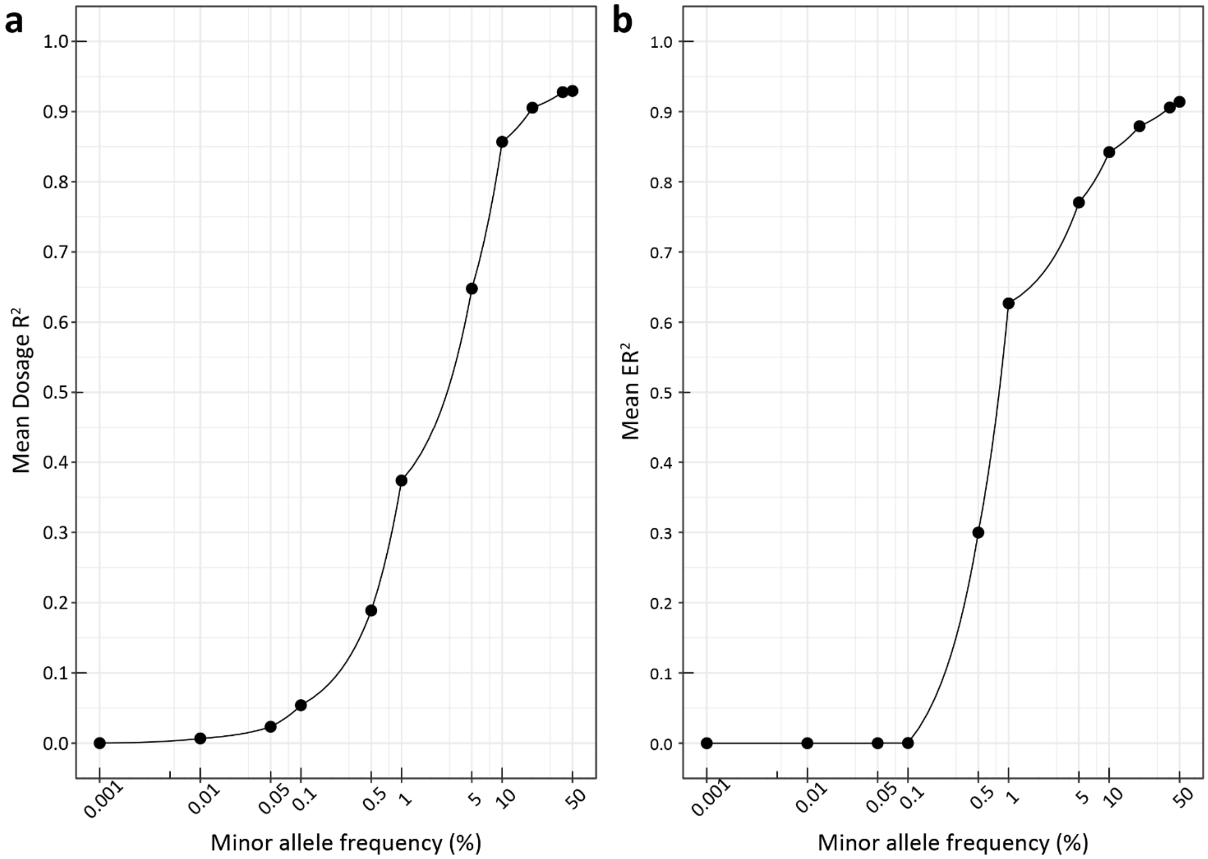

Fig. S5: Imputation Performance. a. Imputation accuracy (Dosage R^2) from Minimac4[10] for all variants (genotyped and ungenotyped) when imputed up to WGS level using the Global Reference Panel. **b.** Imputation accuracy measured using the empirical R^2 (ER^2) metric from Minimac4 of genotyped variants when imputed up to WGS level using the Global Reference Panel.

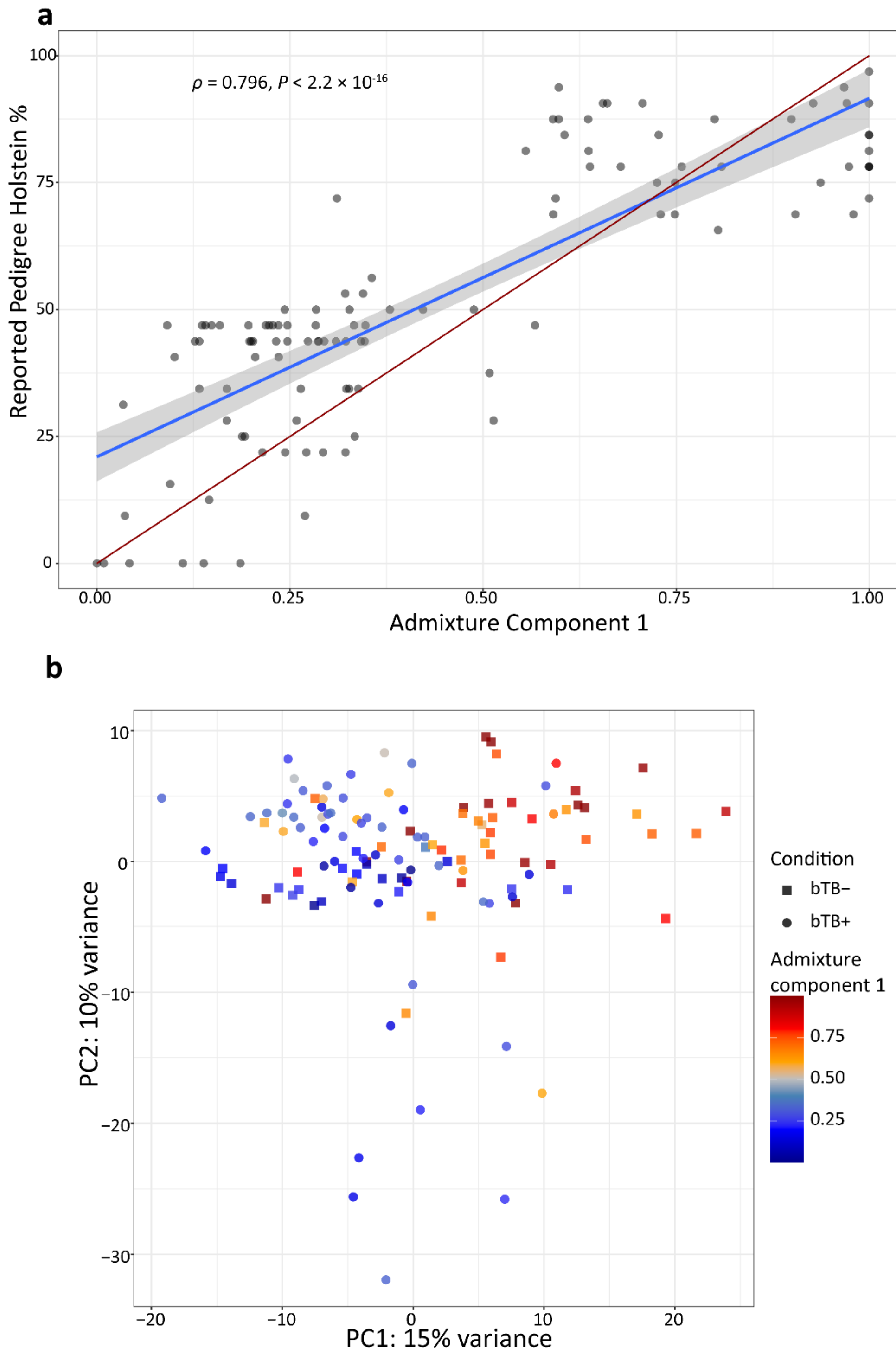

Fig. S6: Admixture analysis and its impact on the transcriptomics principal component analysis (PCA). a Spearman correlation (ρ) between reported pedigree Holstein % and Admixture component 1 from Admixture analysis[14] of 34,272 pruned SNPs. Red line indicates line of parity, blue line indicates line of best fit. **b** Principal component analysis (PCA) of top 1,500 most variable genes for all n = 123 animals after variance stabilising transformation using DESeq2[15]. Principal components PC1 and PC2 are plotted. Animal data points are shaped based on their experimental condition and coloured according to their corresponding Admixture component 1 value.

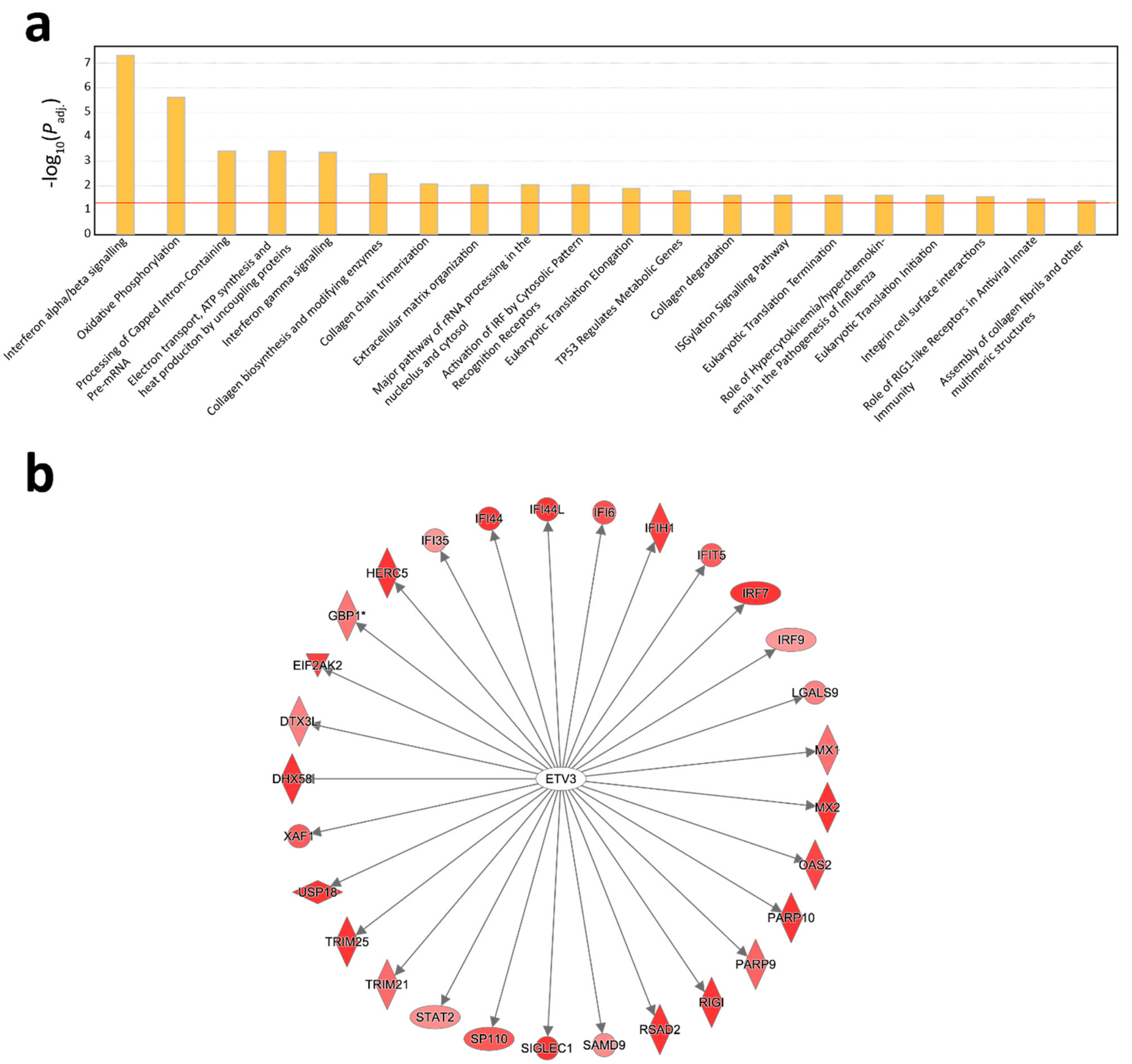

Fig. S7: IPA® enrichment and upstream regulator results for differentially expressed genes. a Bar plot of significantly enriched (FDR $P_{adj.} < 0.05$) Ingenuity® Pathway Analysis (IPA) pathways for highly significant (FDR $P_{adj.} < 0.01$) differentially expressed genes used as input. Bars are ordered corresponding to their $-\log_{10} P_{adj.}$ value. **b** Top upstream biological regulator (ETV3) predicted to be upregulated by IPA based on the highly significant DE genes used as input.

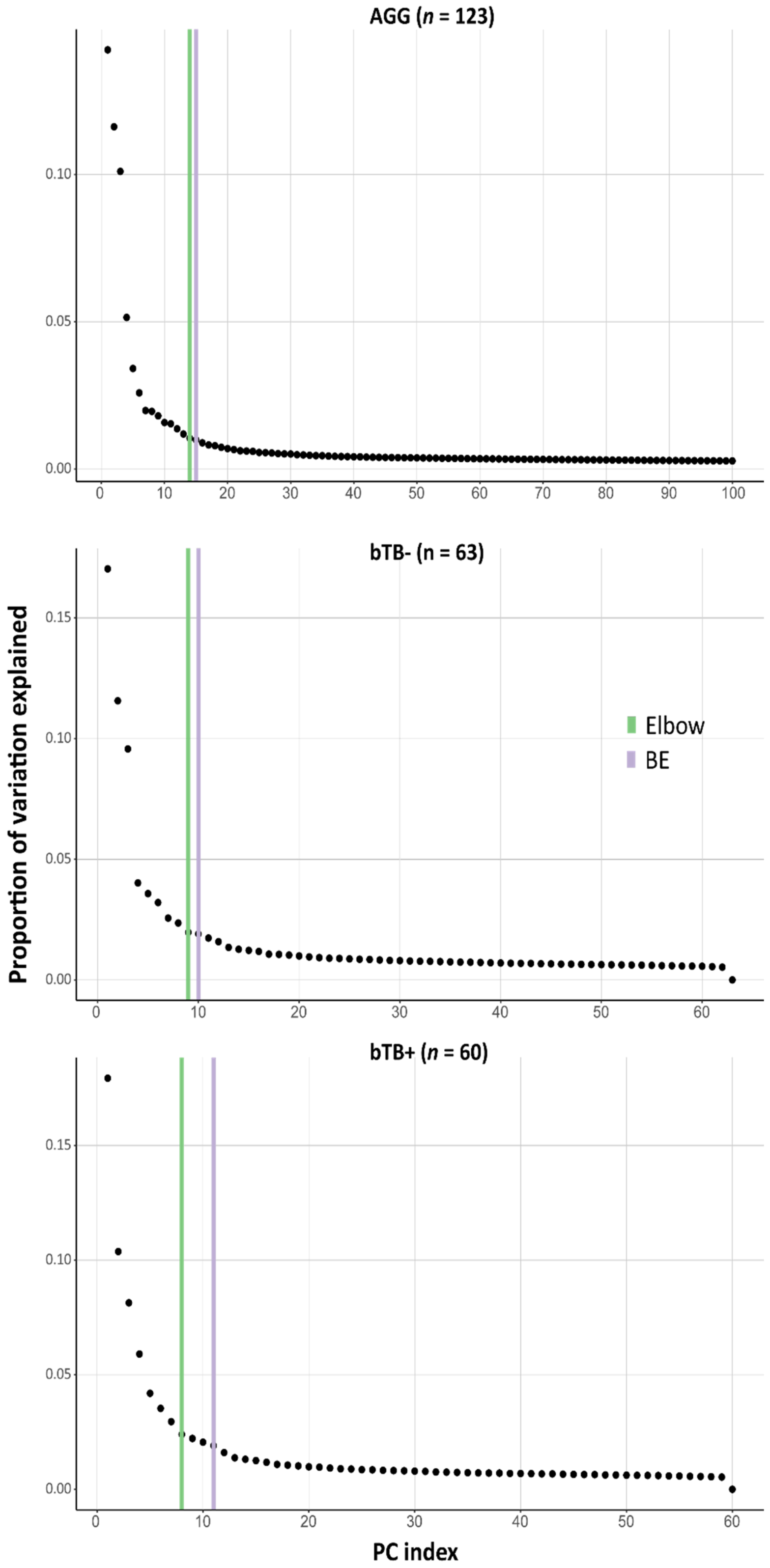

Fig. S8: Transcriptomics principal components (PCs) used in the eQTL analysis. Scree plot showing the proportion of variation explained by each of the transcriptomics PCs identified using the PCAForQTL R package[16] across the all animals group (AGG), the control group (bTB−), and the reactor group (bTB+),

respectively. Green lines correspond to the number of transcriptomic PCs inferred by the elbow method. Purple line denotes the number of PCs inferred by the Buja and Eyuboglu (BE) algorithm[17].

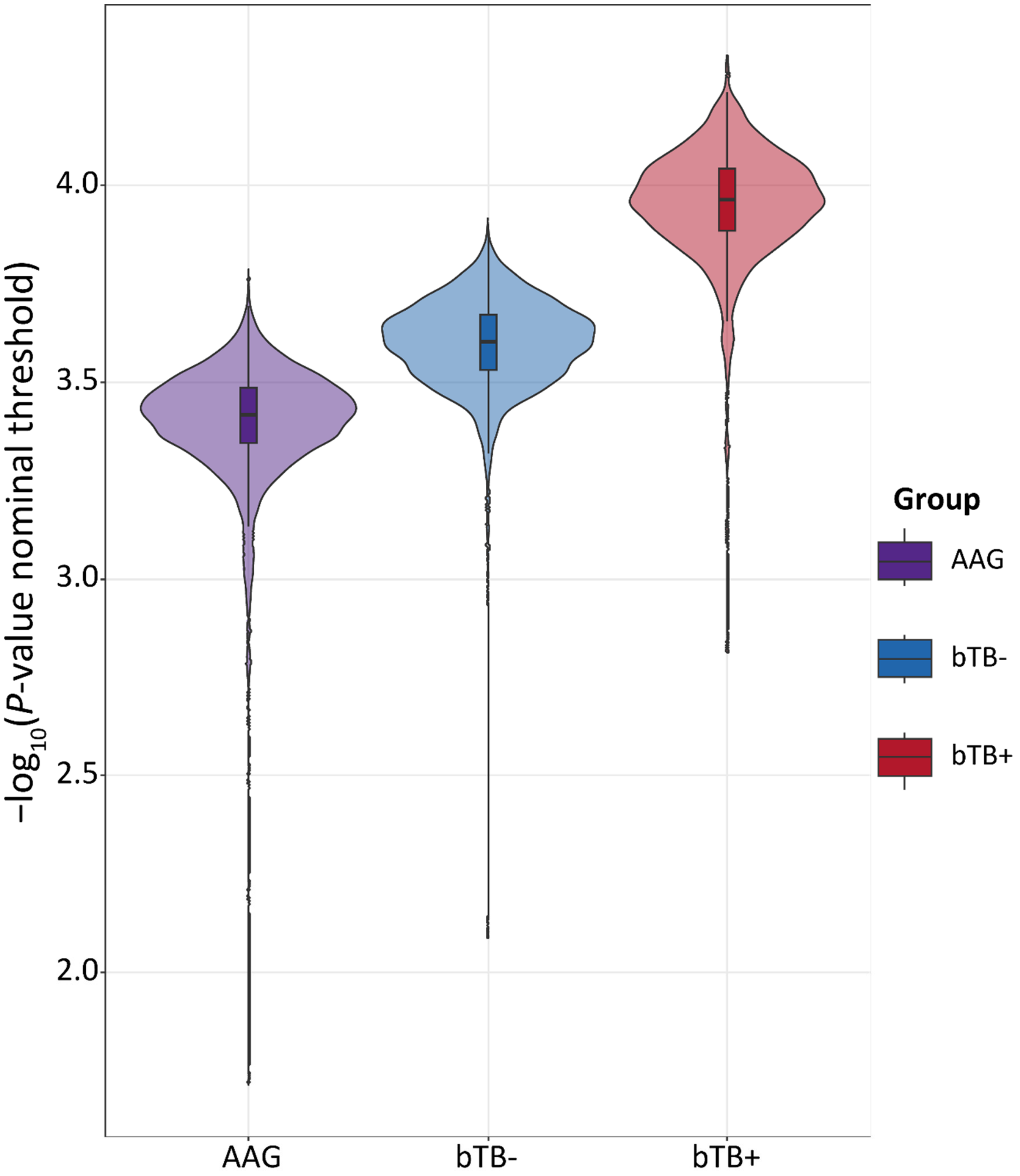

Fig. S9: Nominal P-value thresholds within each group for the determination of *cis*-eVariants. Violin plots showing the distribution of nominal *P*-value threshold per *cis*-eGene for bTB− (control), bTB+ (reactor), and AAG (all animal) groups, respectively.

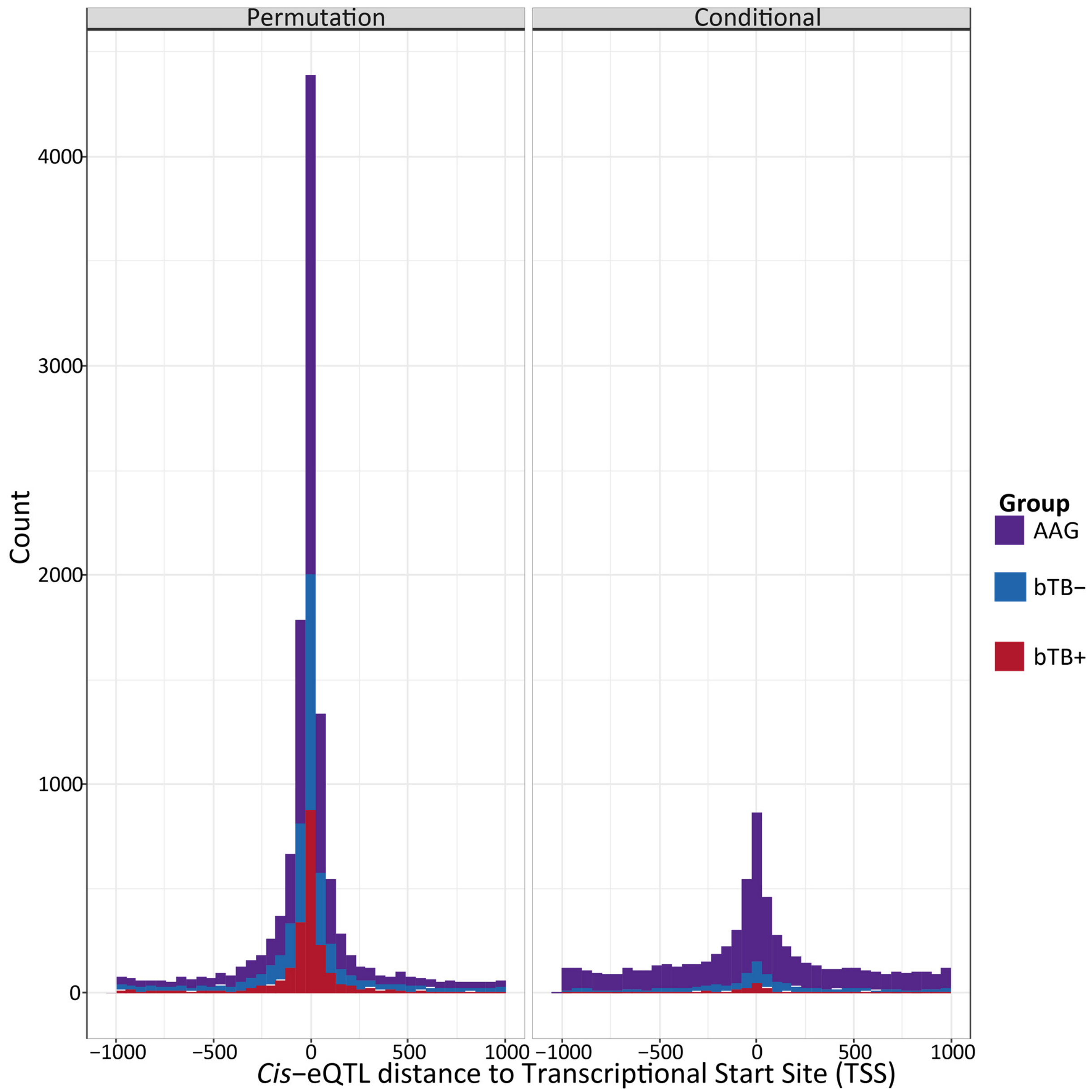

Fig. S10: Distance to transcriptional start site of *cis*-eQTLs. Comparison of the distances to transcriptional start site of all cis-eQTLs identified via the permutation and conditional analysis across the control (bTB−), reactor (bTB+), and combined all animals (AAG) cohorts.

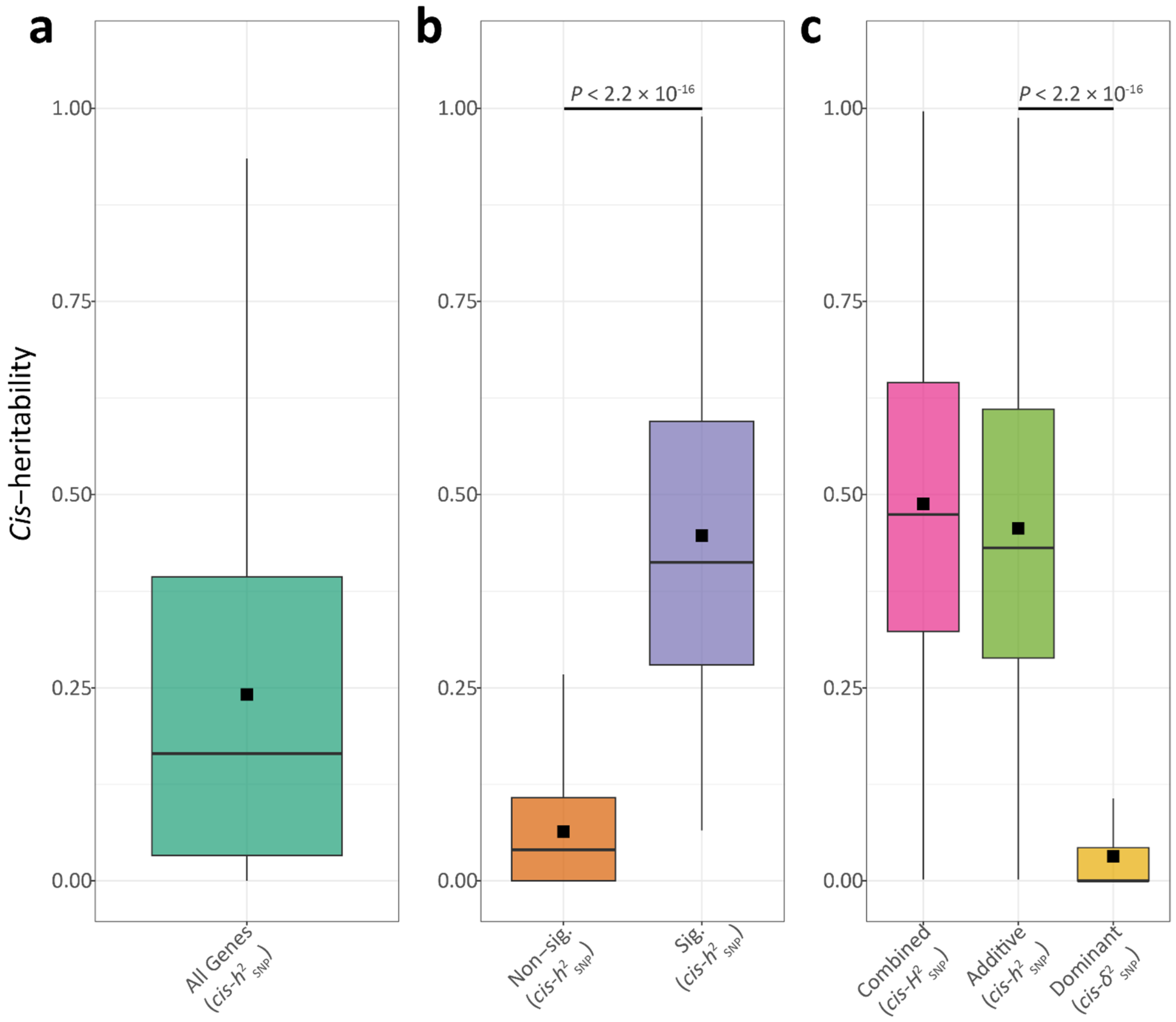

Fig. S11: *Cis*-heritability of gene expression is predominantly governed by additive genetic variance. a Distribution of narrow-sense *cis*-heritability (*cis*-h^2) estimates for all genes (n = 14,563) tested in the *cis*-eQTL analysis in the all animal group (AAG) cohort and for which a GRM could be constructed for. **b** Distribution of *cis*-h^2 values for genes not considered significantly heritable (Non-sig.; likelihood ratio test (LRT) $P_{adj.} > 0.05$) (n = 7,806) and for those considered significantly heritable (Sig.; LRT $P_{adj.} < 0.05$) (n = 6,757). **c** Distribution of broad-sense heritability (H^2_{SNP}) estimates for 5,863 significantly heritable genes (dark-green) with this estimate decomposed into the proportion explained by additive variance (light-green) and dominance variance (δ^2_{SNP}; orange). The box plots in **a, b** and **c** cover the interquartile range with the median line denoted at the centre and mean being denoted by the black square box. The whiskers extend to the most extreme data point that is no more than 1.5 × IQR from the edge of the box. *P*-values in figures **b** and **c** are inferred from the Wilcoxon rank-sum test between the heritability estimates within each group

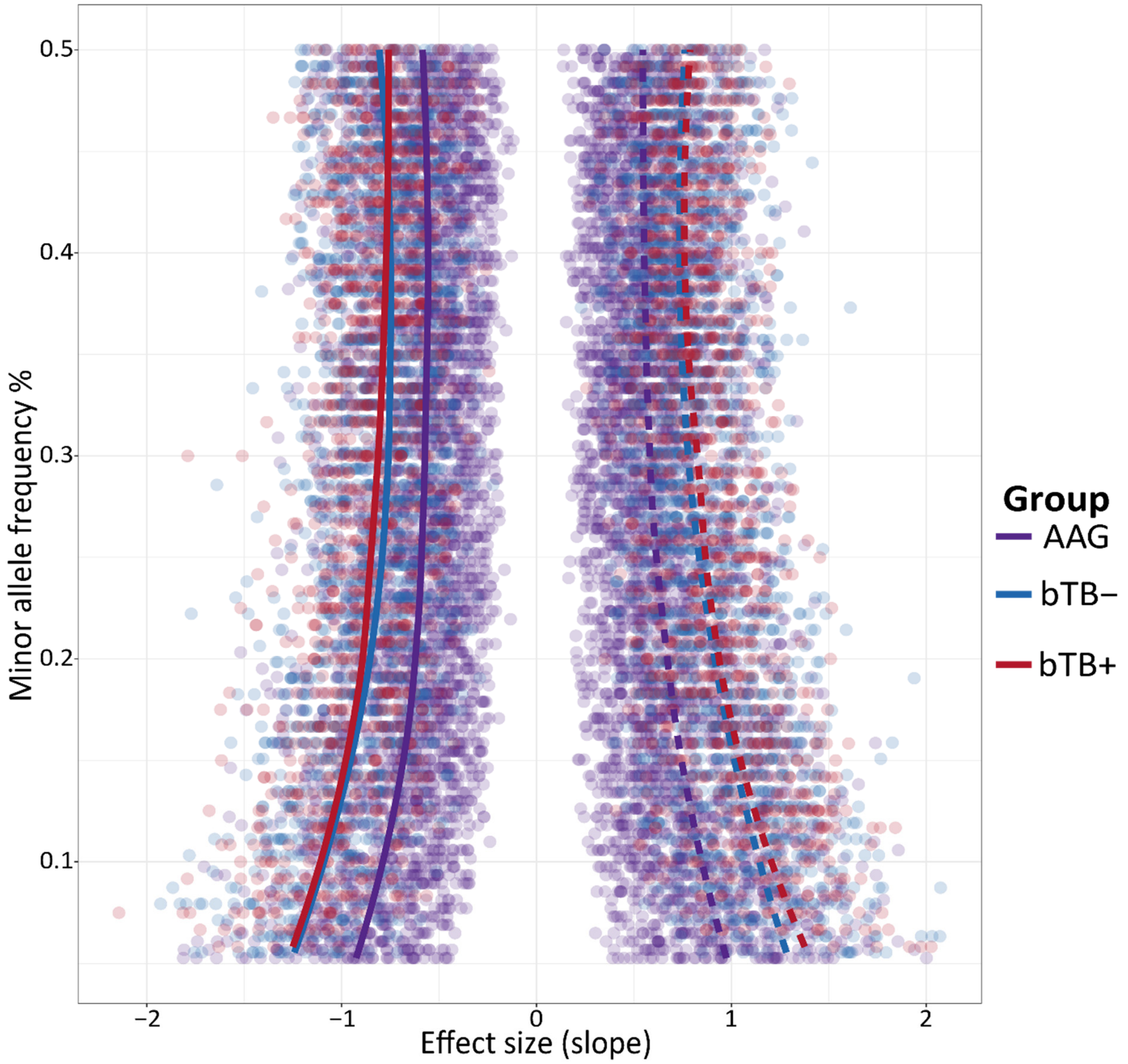

Fig. S12: Visualisation of *cis*-eQTL effect size and relationship to minor allele frequency (MAF). Solid trend lines indicate *cis*-eQTLs where the effect size was negative and dashed lines indicate those where the effect size was positive and were generated using the *geom_smooth* function in R setting the method to "*loess*" in ggplot2.

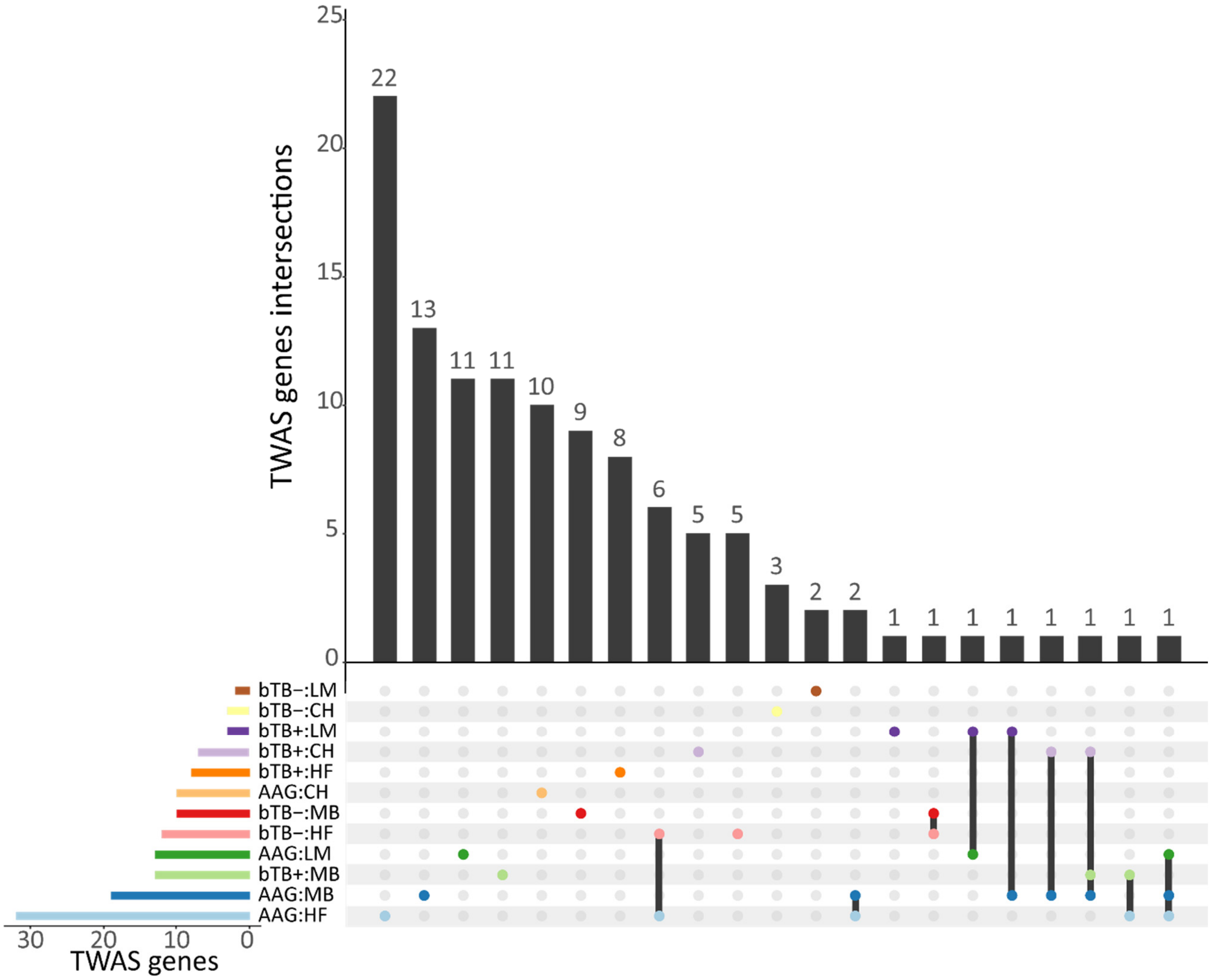

Fig. S13: Overlap of significant TWAS genes across all 12 TWAS groups. Upset plot showing the number and intersection of significant transcriptome-wide association study (TWAS) genes (Bonferroni $P_{adj.} < 0.05$ and $P < 0.05$ post-permutation) across the bTB−, the bTB+, and AAG reference panels identified using the Charolais (CH), Limousin (LM), Holstein-Friesian (HF), and Multi-breed (MB) GWAS data sets.

References

1 Rosen, B. D. *et al. De novo* assembly of the cattle reference genome with single-molecule sequencing. *Gigascience* **9** (2020). https://doi.org/10.1093/gigascience/giaa021

2 Lawrence, M., Gentleman, R. & Carey, V. rtracklayer: an R package for interfacing with genome browsers. *Bioinformatics* **25**, 1841-1842 (2009). https://doi.org/10.1093/bioinformatics/btp328

3 Verma, S. S. *et al.* Imputation and quality control steps for combining multiple genome-wide datasets. *Front. Genet.* **5**, 370 (2014). https://doi.org/10.3389/fgene.2014.00370

4 Winkler, T. W. *et al.* Quality control and conduct of genome-wide association meta-analyses. *Nat. Protoc.* **9**, 1192-1212 (2014). https://doi.org/10.1038/nprot.2014.071

5 Dutta, P. *et al.* Whole genome analysis of water buffalo and global cattle breeds highlights convergent signatures of domestication. *Nat. Commun.* **11**, 4739 (2020). https://doi.org/10.1038/s41467-020-18550-1

6 Riggio, V. *et al.* Assessment of genotyping array performance for genome-wide association studies and imputation in African cattle. *Genet. Sel. Evol.* **54**, 58 (2022). https://doi.org/10.1186/s12711-022-00751-5

7 Danecek, P. *et al.* The variant call format and VCFtools. *Bioinformatics* **27**, 2156-2158 (2011). https://doi.org/10.1093/bioinformatics/btr330

8 Manichaikul, A. *et al.* Robust relationship inference in genome-wide association studies. *Bioinformatics* **26**, 2867-2873 (2010). https://doi.org/10.1093/bioinformatics/btq559

9 Browning, B. L., Tian, X., Zhou, Y. & Browning, S. R. Fast two-stage phasing of large-scale sequence data. *Am. J. Hum. Genet.* **108**, 1880-1890 (2021). https://doi.org/10.1016/j.ajhg.2021.08.005

10 Das, S. *et al.* Next-generation genotype imputation service and methods. *Nat. Genet.* **48**, 1284-1287 (2016). https://doi.org/10.1038/ng.3656

11 Danecek, P. *et al.* Twelve years of SAMtools and BCFtools. *Gigascience* **10** (2021). https://doi.org/10.1093/gigascience/giab008

12 Purcell, S. *et al.* PLINK: a tool set for whole-genome association and population-based linkage analyses. *Am. J. Hum. Genet.* **81**, 559-575 (2007). https://doi.org/10.1086/519795

13 Fort, A. *et al.* MBV: a method to solve sample mislabeling and detect technical bias in large combined genotype and sequencing assay datasets. *Bioinformatics* **33**, 1895-1897 (2017). https://doi.org/10.1093/bioinformatics/btx074

14 Alexander, D. H. & Lange, K. Enhancements to the ADMIXTURE algorithm for individual ancestry estimation. *BMC Bioinformatics* **12**, 246 (2011). https://doi.org/10.1186/1471-2105-12-246

15 Love, M. I., Huber, W. & Anders, S. Moderated estimation of fold change and dispersion for RNA-seq data with DESeq2. *Genome Biol.* **15**, 550 (2014). https://doi.org/10.1186/s13059-014-0550-8

16 Zhou, H. J., Li, L., Li, Y., Li, W. & Li, J. J. PCA outperforms popular hidden variable inference methods for molecular QTL mapping. *Genome Biol.* **23**, 210 (2022). https://doi.org/10.1186/s13059-022-02761-4

17 Buja, A. & Eyuboglu, N. Remarks on Parallel Analysis. *Multivariate Behav Res* **27**, 509-540 (1992). https://doi.org/10.1207/s15327906mbr2704_2
