# Supplementary material for: Integrative genomics sheds light on the immunogenetics of tuberculosis in cattle
Source: Commun Biol. 2025 Mar 24;8:479. doi: 10.1038/s42003-025-07846-x (PMC11933339; doi:10.1038/s42003-025-07846-x)
Supplement: Supplementary file 2 — Description of Additional Supplementary Files [file 42003_2025_7846_MOESM2_ESM.docx]

Description of Additional Supplementary Files

**File name:** Supplementary Data 1

**Description:** Supplementary Tables described in the paper.

**File name:** Supplementary Data 2

**Description:** The source data behind Fig. 2.

**File name:** Supplementary Data 3

**Description:** The source data behind Fig. 3.

**File name:** Supplementary Data 4

**Description:** The source data behind Fig. 4.

**File name:** Supplementary Data 5

**Description:** The source data behind Fig. 5.
