# Supplementary material for: Integrative genomics sheds light on the immunogenetics of tuberculosis in cattle
Source: Commun Biol. 2025 Mar 24;8:479. doi: 10.1038/s42003-025-07846-x (PMC11933339; doi:10.1038/s42003-025-07846-x)
Supplement: Supplementary file 8 — Reporting summary [file 42003_2025_7846_MOESM8_ESM.pdf]

nature portfolio

Corresponding author(s): David MacHugh and Stephen Gordon

Last updated by author(s): Feb 16, 2025

Reporting Summary

Nature Portfolio wishes to improve the reproducibility of the work that we publish. This form provides structure for consistency and transparency in reporting. For further information on Nature Portfolio policies, see our Editorial Policies and the Editorial Policy Checklist.

Statistics

For all statistical analyses, confirm that the following items are present in the figure legend, table legend, main text, or Methods section.

n/a	Confirmed	
☐	☒	The exact sample size (*n*) for each experimental group/condition, given as a discrete number and unit of measurement
☐	☒	A statement on whether measurements were taken from distinct samples or whether the same sample was measured repeatedly
☐	☒	The statistical test(s) used AND whether they are one- or two-sided<br>*Only common tests should be described solely by name; describe more complex techniques in the Methods section.*
☐	☒	A description of all covariates tested
☐	☒	A description of any assumptions or corrections, such as tests of normality and adjustment for multiple comparisons
☐	☒	A full description of the statistical parameters including central tendency (e.g. means) or other basic estimates (e.g. regression coefficient) AND variation (e.g. standard deviation) or associated estimates of uncertainty (e.g. confidence intervals)
☐	☒	For null hypothesis testing, the test statistic (e.g. *F*, *t*, *r*) with confidence intervals, effect sizes, degrees of freedom and *P* value noted<br>*Give P values as exact values whenever suitable.*
☒	☐	For Bayesian analysis, information on the choice of priors and Markov chain Monte Carlo settings
☒	☐	For hierarchical and complex designs, identification of the appropriate level for tests and full reporting of outcomes
☐	☒	Estimates of effect sizes (e.g. Cohen's *d*, Pearson's *r*), indicating how they were calculated

Our web collection on statistics for biologists contains articles on many of the points above.

Software and code

Policy information about availability of computer code

Data collection	Standard code from sequencing providers (Affymetrix and Illumina) was used to gather array and sequencing data respectively.
Data analysis	Version numbers and references for all software and scripts used to process and analyse data are given in the manuscript. The following software were used:<br>USDA-NAGRP data repository (www.animalgenome.org/repository/cattle/UMC_bovine_coordinates), Axiom™ Genome-Wide BOS-1 Array master annotation file (www.thermofisher.com/order/catalog/product/sec/assets?url=TFS-Assets/LSG/Support-Files/Axiom_GW_Bos_SNP_1-na35-annot-csv.zip), USDA-NAGRP BOS-1 ARS-UCD1.2 reference allele file (www.animalgenome.org/repository/cattle/UMC_bovine_coordinates), AnimalTFDB (v.4.0 http://bioinfo.life.hust.edu.cn/AnimalTFDB4/#/), Ingenuity Pathway Analysis (IPA) (Summer 2023 release; Qiagen, https://digitalinsights.qiagen.com/products-overview/discovery-insights-portfolio/analysis-and-visualization/qiagen-ipa/), R (v.4.3.2), python (3.11.4), Axiom Analysis Suite software tool (v 5.1.1.1), ADMIXTURE (v1.3), pophelper(v2.3.1), Plink (v1.9), Beagle (v5.4), Minimac3 (v2.0.1), Minimac4 (v1.0.3), FastQC (v0.11.5), STAR (v2.7.1a), featureCounts (v2.0.0), DESeq2 (v1.40.2), EdgeR (v3.42.5), Tidyverse (v2.0.0), Data.table(v1.14.8), UpSetR(v1.4.0), g:profiler2(v0.2.2), qvalue(v2.32.0), ggplot2(v3.4.4), ggridges(v0.5.4), ggrepel(v0.9.3), Circos (v0.69.9), Circlize(v0.4.15) Vcftools (0.1.16), Bcftools (1.10.2), Htslib (v1.10.2), PCAForQTL (v.0.1.0), TensorQTL (v1.0.8), RNOmni(v1.0.1), vcfR (v1.14.0), devtools(v2.4.5), SNPRelate(v1.34.1), Bigsnpr (v1.10.8), Bigstatsr (v1.5.6), GCTA (v1.94.1), MOSTWAS (v.0.1.0) , eGene_detection.R (https://github.com/FarmGTEx/PigGTEx-Pipeline-v0/blob/master/08_molQTL_mapping/TensorQTL/eGene_detection.R), parquet2txt.py (https://github.com/FarmGTEx/PigGTEx-Pipeline-v0/blob/master/08_molQTL_mapping/TensorQTL/parquet2txt.py), aFC.py (https://github.com/secaste/aFC), 03_SNP_calling.smk (https://github.com/FarmGTEx/PigGTEx-Pipeline-v0/blob/master/02_RNA-Seq/03_SNP_calling.smk), GATK (v 4.3.0.0), GCTA (v. 1.94.0) lmtest R package (v. 0.9.40)

For manuscripts utilizing custom algorithms or software that are central to the research but not yet described in published literature, software must be made available to editors and reviewers. We strongly encourage code deposition in a community repository (e.g. GitHub). See the Nature Portfolio guidelines for submitting code & software for further information.

Data

Policy information about availability of data

All manuscripts must include a data availability statement. This statement should provide the following information, where applicable:

- Accession codes, unique identifiers, or web links for publicly available datasets
- A description of any restrictions on data availability
- For clinical datasets or third party data, please ensure that the statement adheres to our policy

RNA sequencing data for all 123 animals are available on the GEO database with the Bioproject Accession GSE255724. Comprehensive metadata for all samples are available in Supplementary Table 1. Raw and normalised gene expression matrices for the differential expression and eQTL analyses (for each specific cohort) are available under GEO accession number GSE255724. Raw SNP-array and filtered imputed WGS data for all 123 animals in addition to raw cis-eQTL results, TWAS expression models and GWAS data for the four breed cohorts (CH,HF,LM,MB) are available at Zenodo. The Ensembl gene annotation(release 110) is available at https://ftp.ensembl.org/pub/release-110/gtf/bos_taurus/. The imputation reference panel is available from Dutta et al., 2020. Raw M. bovis infection susceptibility GWAS datasets are available from Ring et al., 2019.

Research involving human participants, their data, or biological material

Policy information about studies with human participants or human data. See also policy information about sex, gender (identity/presentation), and sexual orientation and race, ethnicity and racism.

Reporting on sex and gender	na
Reporting on race, ethnicity, or other socially relevant groupings	na
Population characteristics	na
Recruitment	na
Ethics oversight	na

Note that full information on the approval of the study protocol must also be provided in the manuscript.

Field-specific reporting

Please select the one below that is the best fit for your research. If you are not sure, read the appropriate sections before making your selection.

☒ Life sciences ☐ Behavioural & social sciences ☐ Ecological, evolutionary & environmental sciences

For a reference copy of the document with all sections, see nature.com/documents/nr-reporting-summary-flat.pdf

Life sciences study design

All studies must disclose on these points even when the disclosure is negative.

Sample size	No statistical method was applied to predetermine sample size. Our sample size is in the range of typical eQTL studies. Cattle breeds such as Holstein have a low effective population size (Ne ~ 100) and as a result, less individuals are required to capture genetic diversity than in e.g., human populations
Data exclusions	Rare alleles (minor allele frequency < 0.05), alleles which deviated from Hardy Weinberg Equilibrium (HWE P < 0.000001) and those with a dosage R2 value < 0.6 were excluded from the eQTL analysis.
Replication	This was an exploratory study with the objective of investigating the transcriptional differences between control and M. bovis infected cattle. No replication was applied. Replication would involve collecting a similar number of samples which would be logistically challenging and costly
Randomization	No randomisation took place. Cattle were experimentally designated based on their reaction to the single intradermal comparative tuberculin test (SICTT) as bTB+ (if the reaction was positive) or bTB- (if the reaction was negative).
Blinding	Blinding was not required for this study as this experiment did not involve the administration of drugs/therapeutics to the animals. The study was exploratory in nature to assess the genomic architecture underpinning the transcriptional differences between bTB- and bTB+ cattle, respectively.

Reporting for specific materials, systems and methods

We require information from authors about some types of materials, experimental systems and methods used in many studies. Here, indicate whether each material, system or method listed is relevant to your study. If you are not sure if a list item applies to your research, read the appropriate section before selecting a response.

Materials & experimental systems

n/a	Involved in the study
☒	☐ Antibodies
☒	☐ Eukaryotic cell lines
☒	☐ Palaeontology and archaeology
☐	☒ Animals and other organisms
☒	☐ Clinical data
☒	☐ Dual use research of concern
☒	☐ Plants

Methods

n/a	Involved in the study
☒	☐ ChIP-seq
☒	☐ Flow cytometry
☒	☐ MRI-based neuroimaging

Animals and other research organisms

Policy information about studies involving animals; ARRIVE guidelines recommended for reporting animal research, and Sex and Gender in Research

Laboratory animals	Laboratory animals were not used.
Wild animals	This study did not involve wild animals
Reporting on sex	Only male cattle were considered. There are two reasons for this. Firstly, the bTB reactor (bTB+) cohort of cattle that are maintained for diagnostics potency testing at the Irish Department of Agriculture Food and the Marine (DAFM) research facilities (Backweston, Co. Kildare, Irealnd) are all male. Secondly, cattle breeding in Ireland and many other countries focuses on genetic improvement through artificial insemination (AI) based breeding programmes.
Field-collected samples	The diseased cohort (bTB+) of animals were collected from the Irish Department of Agriculture Food and the Marine (DAFM) animal research facility (Backweston, Co. Kildare, Ireland). These cattle were maintained in standard housing. The control animals were collected from the UCD experimental farm (Lyons, Co. Kildare, Ireland) and from neighboring farms without a history of M. bovis infection.
Ethics oversight	All experimental procedures involving animals were conducted under ethical approval from the University College Dublin (UCD) Animal Research Ethics Committee (AREC-19-09-MacHugh) and experimental license AE18982/P141 from the Irish Health Products Regulatory Authority (HPRA) in accordance with the Cruelty to Animals Act 1876 and in agreement with the European Union (Protection of Animals Used for Scientific Purposes) regulations 2012 (S.I. No.543 of 2012).

Note that full information on the approval of the study protocol must also be provided in the manuscript.

Plants

Seed stocks	na
Novel plant genotypes	na
Authentication	na
